# Unraveled role of TLR7-mediated interferon signaling activation in COVID-19

**DOI:** 10.3389/fcimb.2025.1658249

**Published:** 2025-10-08

**Authors:** Junjian Xue, Xiaoyin Wang, Hui Wang, Bin Qiao, Pengfei Gao, Bin Ren, Shushan Yan

**Affiliations:** ^1^ Department of Gastrointestinal and Anal Diseases Surgery, The Affiliated Hospital of Shandong Second Medical University, Weifang, China; ^2^ Operating Room, The Affiliated Hospital of Shandong Second Medical University, Weifang, China

**Keywords:** toll-like Receptor 7, interferon, SARS-CoV-2, T cell immunity, B cell immunity, post-acute sequelae of SARS-CoV-2 infection

## Abstract

Emerging evidence underscores the critical role of Toll-like receptor 7 (TLR7)-mediated interferon (IFN) signaling in host defense against viral infections including SARS-CoV-2, through the modulation of both innate and adaptive immunity. However, the specific mechanisms by which TLR7 activation shapes SARS-CoV-2-specific immune responses, particularly via IRF-IFN pathways, remain incompletely elucidated. This review synthesizes current findings on how intrinsic TLR7-driven IFN signaling influences viral clearance, modulates adaptive immunity, and contributes to autoantibody production in COVID-19. A deeper understanding of these processes is essential for developing targeted therapeutic interventions and improved vaccines aimed at mitigating severe COVID-19 and preventing post-acute sequelae of SARS-CoV-2 infection (PASC).

## 1 Introduction

Coronavirus disease 2019 (COVID-19), caused by severe acute respiratory syndrome coronavirus 2 (SARS-CoV-2) virus, has emerged as a formidable global challenge, transcending immediate health crises to encompass multifaceted long-term implications (Zhu et al., 2020). The ongoing battle against SARS-CoV-2 infection has spurred in-depth exploration into the complex mechanisms of virus-host interactions, particularly focusing on host immunity. SARS-CoV-2 infection facilitates a cascade of inflammatory and immune responses by activating interferons (IFNs) and Toll-like receptors (TLRs), which are implicated in shaping the clinical outcomes of COVID-19, spanning asymptomatic carriage to acute respiratory distress syndrome (ARDS), multi-organ failure, and other severe manifestations (Paludan et al., 2021). As pattern recognition receptors (PRRs), TLRs function as a bridge linking innate immunity with adaptive immunity by recognizing conserved pathogen-associated molecular patterns (PAMPs) (Akira et al., 2006; Kawai and Akira, 2008). Among them, TLR2 and TLR4 are well documented TLRs contributing to the host defense against COVID-19 (Ashhurst et al., 2022; Sahanic et al., 2023; Habeichi et al., 2024). As a double-stranded RNA (dsRNA) sensor, TLR3 can also sense dsRNA intermediate during replication of single-stranded RNA (ssRNA) in host cells. Reduced expression of peripheral blood TLR3 is linked to an adverse outcome in severe COVID-19 patients, suggesting an underlying role of TLR3 in regulating IFN response during SARS-CoV-2 infection (Menezes et al., 2021). A number of studies have also demonstrated that other endosomal TLRs play important roles in the onset and progression of COVID-19, including TLR7, TLR8, and TLR9 (Fitzgerald and Kagan, 2020; Manik and Singh, 2022). *Tlr7* is a gene located on the X chromosome. TLR7 is a crucial sensor in SARS-CoV-2 infection because it recognizes viral ssRNAs. This recognition triggers innate immune responses, including the production of type I IFNs, which are essential for controlling early viral replication and shaping adaptive immunity. Emerging evidence has supported the critical role of endosomal TLR7 and TLR7-mediated IFN signaling activation in SARS-CoV-2 infection, particularly regarding the modifying effects on the innate immune reaction initiation, cytokine storm, and systemic immune disorders in COVID-19. Understanding the role and mechanism of TLR7-mediated IFNs signaling activation in viral immunity is of utmost importance in exploring more effective strategy for the control of SARS-CoV-2 infection.

Long-COVID affects at least 10% of SARS-CoV-2 infections. Increasing studies have implicated that long-COVID is characteristic of a sustained proinflammatory immune profile, compromised humoral immunity, and diminished cellular immunity (Woodruff et al., 2022; Klein et al., 2023). Despite research progress during the past few years, the etiology and immunopathological mechanism underlying post-acute sequelae of SARS-CoV-2 infection (PASC) remain not fully understood due to the heterogeneous nature of the condition, which presents with a wide spectrum of symptoms and involves multiple organ systems. Furthermore, the underlying mechanisms may involve persistent viral reservoirs, autoimmune responses, and chronic inflammation, all of which are complex, interconnected, and difficult to disentangle in human studies. It has been well documented that the intrinsic TLR7 can induce a prominent type-I IFN response and robust antiviral immune responses by regulating adaptive immunity against SARS-CoV-2 infection (Rossmann et al., 2021; Miquel et al., 2023). Nonetheless, the underlying mechanism of TLR7 in regulating the adaptive immune response and its interaction with IFN signaling in COVID-19 and long-COVID remains not fully understood. The specific role of TLR7 in shaping the adaptive immune response to SARS-CoV-2, particularly its synergy with or antagonism of IFN signaling, remains unclear due to the complexity of the cytokine environment. This is compounded in long-COVID by the challenge of differentiating between ongoing viral persistence and virus-induced autoimmune pathology. Furthermore, most mechanistic insights come from murine models, which do not fully recapitulate the human immune response to the virus. This review is aimed to summarize currently published studies on the potential roles and mechanisms of TLR7 and IFN signaling in COVID-19. Elucidating the possible mechanisms behind innate and adaptive immune responses of TLR7-mediated IFN signaling activation in SARS-CoV-2 infection is promising for the exploration of more effective vaccines and therapeutic strategies particularly for severe COVID-19 and long-COVID.

## 2 Clinical evidence supporting the critical role of intrinsic TLR7 and TLR7 mutation in COVID-19

SARS-CoV-2 has a positive-sense, single-stranded, linear RNA genome. The ssRNA genome structure laid the foundation for the importance of nucleic acid-TLRs interactions. Many clinical cases have supported the importance of them in COVID patients, particularly TLR7. COVID-19 patients with the functional variants of TLR7 have an impaired type-I IFN response in the peripheral circulation, characterized by significant downregulation of IRF7, IFNβ1, IFNγ and IFN-stimulated gene 15 (ISG15) (Fallerini et al., 2021). The COVID-19 pandemic exhibits a male sex bias, with males being admitted to the intensive care unit (ICU) 2.84 times more frequently, and experiencing a 1.39 times higher mortality rate compared to females (Spiering and de Vries, 2021). *Tlr7* gene has a gene-dose effect caused by X chromosome inactivation (XCI) escape. Recent studies have proposed that the gene-dose of *Tlr7* may play a vital role in the sex-skewed severity observed in COVID-19 (Gómez-Carballa et al., 2022). Young men with severe COVID-19 exhibit the *Tlr7* genetic variants, including the X-linked recessive TLR7 deficiency (van der Made et al., 2020; Asano et al., 2021). Approximately 1% of men under 60 years old with life-threatening COVID-19 exhibit X-linked recessive *Tlr7* deficiency (Asano et al., 2021). In male COVID-19 patients, rare variants of Arg920Lys and Asp41Glu in *Tlr7* gene have been identified, leading to functional impairment and the downregulation of cytokine-mediated signaling activation (Mantovani et al., 2022). Males with loss-of-function variants in *TLR7* have an increased risk of life-threatening COVID-19 pneumonia. This suggests that gender disparities in SARS-CoV-2 outcomes result from the combined effects of multiple factors, including differences in immune responses and sex hormones (Vikse et al., 2020; Asano et al., 2021; Vadakedath et al., 2021; Naushad et al., 2023; Zovi et al., 2023). Besides, in the Korean population, no significant differences are observed in the genotype or allele frequencies of *Tlr7* rs864058 polymorphism between female patients with COVID-19 and the healthy controls, supporting that the genetic polymorphisms of *Tlr7* rs864058 is of low frequency and not associated with SARS-CoV-2 infection in Korean females (Zayed et al., 2023). Moreover, loss-of-function variants of *Tlr7* have also been highlighted in modulating the long-term complications of post-COVID-19, such as severe long-term neurological deterioration, brain fog, and hypoxemic pneumonia (Bagci et al., 2023; Noor Eddin et al., 2023). In conclusion, *Tlr7* gene mutation plays a pivotal role both in the acute antiviral response, disease severity, and the long-term immunity against COVID-19 in a sex-skewed manner. A notable phenomenon of XCI escape occurs in immune cells, with the escape of the *Tlr7* gene in pDCs being particularly prominent. Escape frequencies are influenced by genetic background, environmental factors, and epigenetic regulation. A deeper examination of tissue-specific escape patterns, particularly in immune cell subsets, and the role of epigenetic modifiers such as XIST regulators or chromatin accessibility would provide a more robust framework. Furthermore, situating TLR7 within a broader X-chromosomal regulatory network, including interactions with other escapee genes like TLR8 or CXCR3 (Du et al., 2025; Sakkas et al., 2025), could reveal synergistic immunopathological pathways and improve the translational relevance of the proposed mechanism. In patients with systemic sclerosis (SSc), the frequency of TLR7 escape in pDCs is significantly increased, accompanied by aberrant overexpression of TLR7 and hyperactivation of IFN-I signaling, suggesting a link to autoimmune pathology (Gabriele et al., 2021; Spiering and de Vries, 2021; Du et al., 2025). X-inactive specific transcript (XIST), a key long non-coding RNA (lncRNA) responsible for initiating XCI, mediates chromosomal silencing by recruiting histone modifiers and DNA methyltransferases (Cerase et al., 2021; Rodermund et al., 2021). Its expression is dynamically regulated by culture medium composition, cellular differentiation status, and chromatin remodeling factors such as Chd8. Instability in XIST expression may lead to increased XCI escape. At the chromatin structural level, the *Tlr7* gene dissociates from the suppressive region of the inactive X chromosome (Xi) upon escape and forms a transcriptionally active chromatin loop (Cloutier et al., 2022). Multiple immune-related genes on the X chromosome can cooperatively escape XCI, forming an immunoregulatory network. In addition to TLR7, TLR8 also frequently escapes in pDCs and acts synergistically with TLR7 to enhance the IFN-I response, thereby promoting fibrotic processes in diseases such as SSc (Du et al., 2025; Sakkas et al., 2025). As an adaptor protein in the TLR signaling pathway, escape of TASL further amplifies the downstream signaling of TLR7 (Scofield et al., 2025). Escaping XCI in immune cells, such as pDCs, could lead to biallelic expression of TLR7 and other X-linked immune regulators. This elevated gene dosage would potentially hypersensitize the innate immune system, thereby predisposing it to an exaggerated inflammatory response upon SARS-CoV-2 infection, elevating innate antiviral sensing, amplifying proinflammatory signaling and pushing the immune response toward a pathogenic cytokine storm. While potentially enhancing early virus control, this hyperactive response may predispose individuals to loss of immune balance, which accelerates cytokine-driven pathology and increases the risk of severe immunopathology in COVID-19. To unravel the impacts of gender bias and genetic variants of *Tlr7* during SARS-CoV-2 infection not only provides insight into understanding the mechanism of TLR7 in antiviral immunity, but allows for potential therapeutic target for stimulation of TLR7-mediated IFN signaling activation during antiviral protection.

## 3 Role of TLR7 in COVID-19 by regulating inflammation and autoimmunity

### 3.1 Role of TLR7 and its interaction with other endosomal TLRs during the antiviral innate immunity

TLR7, predominantly expressed in sentinel immune cells such as monocytes, macrophages, and plasmacytoid dendritic cells (pDCs), plays a pivotal role in initiating type I IFN responses against viral infections. It is also functionally significant in B cells, where it promotes spontaneous germinal center formation, autoantibody production, and autoimmune activation (Soni et al., 2014; Cosgrove et al., 2023). TLR7 recognizes the ssRNAs of various viruses, including human immunodeficiency virus 1 (HIV-1), influenza virus, respiratory syncytial virus (RSV), and SARS-CoV-2, thereby activating downstream signaling pathways such as MyD88-dependent IFN production and inflammatory cytokine release (Fitzgerald and Kagan, 2020). While TLR7 has been extensively studied in the context of other viral infections, its role in SARS-CoV-2 immunobiology requires more precise contextualization. For instance, during HIV-1 infection, TLR7 synergizes with Fas/FasL to deplete CD4+ T cells via IFN regulatory factor 5 (IRF5) activation (Carmona-Pérez et al., 2023). In influenza, TLR7/MyD88 signaling promotes NF-κB activation and modulates T-cell responses, though its downregulation appears beneficial for viral control (Shi et al., 2020). Similarly, TLR7 and TLR8 collaborate in Th1-polarized antiviral responses against influenza and HIV (Heil et al., 2004). In RSV infection, TLR7 activation in pDCs and conventional DCs induces IFNβ through MyD88 and IFNAR pathways, while also facilitating IL-33 production in macrophages in coordination with TLR3 (Qi et al., 2015; Kim et al., 2019). Even in DNA viral infections such as EBV, TLR7 modulates IRF7 activation and interferon production, illustrating its broad functional versatility (Martin et al., 2007; Valente et al., 2012). However, a direct comparative framework linking these findings to SARS-CoV-2 remains inadequately developed. Transitions from general virology to COVID-19-specific mechanisms are often abrupt, and the relevance of other viral models (e.g., HIV, RSV, influenza) to SARS-CoV-2 pathogenesis is not systematically justified. A more integrated analysis should highlight both conserved and distinct roles of TLR7 across viruses, particularly within the unique immunopathological environment of SARS-CoV-2 infection.

Emerging evidence underscores the central role of TLR7 in SARS-CoV-2 infection. It mediates type I IFN and proinflammatory cytokine production (e.g., IL-6, TNF-α) via IRF5 activation in dendritic cells (Cords et al., 2023). Impaired TLR7 function in lung DCs and macrophages is linked to severe pulmonary immunopathology (Qin et al., 2021; Wu and Yang, 2021; Huang et al., 2023; Miquel et al., 2023). Additionally, SARS-CoV-2 skews macrophage polarization toward a proinflammatory phenotype and disrupts efferocytosis via coordinated TLR7/STING activation, exacerbating tissue damage and delaying repair (Salina et al., 2022; García-Nicolás et al., 2023). Although TLR7 deficiency in mice attenuates IFN and inflammatory mediator expression, suggesting a protective role for TLR7-dependent IFN responses, the precise mechanisms underlying its dual roles, antiviral protection versus hyperinflammation, remain unclear (Ghimire et al., 2025). The temporal dynamics of TLR7 activation critically influence immune outcomes. Early and transient induction supports controlled IFN-mediated antiviral responses, whereas delayed or persistent signaling often correlates with dysregulated inflammation and impaired viral clearance. However, the protective role of TLR7 against lethal SARS-CoV-2 infection in mice should be interpreted with caution due to the mouse model’s translational limitations and a body of contradictory evidence implicating TLR7 in hyperinflammation. Future studies employing non-human primate (NHP) COVID-19 models, such as rhesus macaques or cynomolgus monkeys, would be highly appropriate and scientifically advantageous for further investigating the role of TLR7 in SARS-CoV-2 infection and the immunopathology. NHPs share closer physiological, immunological, and genetic similarities with humans compared to murine models, particularly in terms of respiratory anatomy, immune cell subsets, Toll-like receptor expression patterns, and disease progression. This makes them especially relevant for studying nuanced and clinically translatable mechanisms of viral immunity, inflammatory regulation, and organ-specific responses, such as those involved in acute COVID-19 pneumonia or long-COVID.

Furthermore, while SARS-CoV-2 affects multiple extrapulmonary organs (e.g., heart, kidney, nervous system) in long-COVID, the tissue-specific regulation of TLR7 and its interactions with other endosomal TLRs remain poorly understood. Future studies should clarify how TLR7 signaling operates across different physiological contexts to reconcile its protective and pathogenic effects in COVID-19.

### 3.2 Dysregulation of adaptive immunity-mediated by TLR7 during SARS-CoV-2 infection

The intrinsic TLR7 has garnered significant attention for its potential therapeutic implications in various viral infections by managing adaptive immunity during the past few years. Influenza A virus leads to imbalanced proportions of Th1/Th2 and Th17/Treg by activating TLR7-IRF3 signaling (Zheng et al., 2021). The study by Carmona-Pérez L and the colleagues has implicated that the activation of TLR7/IRF-5 axis leads to reduced memory CD4^+^T cell in response to HIV-1 infection (Carmona-Pérez et al., 2023). A TLR7/9-IRF7 signaling axis in pDCs has also been documented in the regulation of CD4^+^ T cell response to HIV-1 infection (Chehimi et al., 2010). IRF5-deficient B cells exhibit diminished TLR7 and TLR9 expression and cytokine-induced IgG2a/c class-switch during polyoma virus infection, suggesting a TLR7/9-IRF5 axis in B cell-mediated antiviral immunity (Fang et al., 2012). Taken together, all these findings have strongly supported the critical role of TLR7 and the interaction with other endosomal TLRs in adaptive immunity against viral infections, including Influenza A, HIV-1 and polyoma viruses.

Emerging studies have implicated an uncoordinated adaptive immune response in COVID-19, while the potential molecular mechanism has not been fully understood. SARS-CoV-2 infection induces the dysregulation of T cell response, characterized by the expansion of regulatory T cell population, reduced T effector cell activation and enhanced SARS-CoV-2-specific T cell immunity (Rümke et al., 2023; Yin et al., 2024). An activated Th2- and Th17-associated immunity is observed following SARS-CoV-2 infection via the viral S protein (Jeong et al., 2023; Zhang et al., 2024). Prolonged antigen exposure leads to T cell exhaustion, impairing their ability to effectively recognize and eliminate SARS-CoV-2-infected cells. An imbalance of T-cell immunity has been confirmed in severe COVID-19, characterized by activated Th17 cells, incompetent memory T cells, and increased exhausted T cells (Das et al., 2023; Vazquez-Alejo et al., 2023). Besides, an increased CD8^+^ T cell exhaustion and abnormal NK-like CD16^+^CD8^+^ T cells has been observed in severe COVID-19 patients (Schreibing et al., 2022). Moreover, SARS-CoV-2 may hinder the robust memory B-cell and T-cell response upon re-exposure to viral reservoirs in the infected cells or tissues that harbor persistent viral genetic materials, such as DNAs or RNAs, allowing the virus to evade immune clearance and antiviral therapies. Nevertheless, SARS-CoV-2 vaccines can prevent from severe outcomes of COVID-19 by inducing a sufficient IFN response and a long-lasting and SARS-CoV-2-specific adaptive immune response mediated by CD4^+^ and CD8^+^ T cells (Zollner et al., 2021; Maringer et al., 2022; Moyon et al., 2022). Inadequate or prolonged IFN response contributes to the persistence of clinical symptoms and long-term complications of COVID-19 by feedback regulation of adaptive immunity (Elizalde-Díaz et al., 2022; Di Stadio et al., 2023). Taken together, the impaired immune recognition and immune killing by CD8^+^T cells, inhibited T-cell and B-cell responses, T cell exhaustion, imbalance of Th1/Th2 and Th17/Treg and the damaged IFN response contribute to the systemic inflammation and uncoordinated adaptive immunity in COVID-19 and long-COVID.

Some pharmacological agents (Imidazoquinoline, Thiazoquinolone, etc.) or adjuvants (Resiquimod R848, etc.) for COVID-19 vaccine acting on TLR7 or TLR8 are potentially used to combat COVID-19 by activating TLR7/8 signaling and inducing prominent type I IFN and Th1 antiviral responses, suggesting the potential role of endosomal TLR7/8 in regulating adaptive immunity in COVID-19 (Khalifa and Ghoneim, 2021). In the presence of TLR7/8 agonists, SARS-CoV-2 virus-like particles (VLPs) have been demonstrated to promote high neutralizing antibody titers, prominent type-I IFNs and differentiation of antigen-specific CD8^+^ T cells and Th1-biased CD4^+^ T cells (Salvi et al., 2021; Sebastião et al., 2023). T cell survival, activation and Th1 polarization are induced by SARS-CoV-2 subunit vaccines adding with TLR7/8 agonist as adjuvants (Yan et al., 2023; Yin et al., 2023). Therefore, Th1-mediated adaptive immune response in company with TLR7/8 stimulation is a critical determinant of SARS-CoV-2 vaccine efficacy. Besides, B cell-intrinsic TLR7 is involved in the regulation of B-cell immune response and the production of IgG2b/IgG2c neutralizing antibody in SARS-CoV-2 infection (Miquel et al., 2023). It has been reported that gain-of-function variants of TLR7 drive B cells activation in a B cell-intrinsic manner in systemic autoimmune diseases, while loss-of-function TLR7 variants confer higher risk of severe COVID-19 and poor prognosis (Cosgrove et al., 2023; Punnanitinont et al., 2023). Accordingly, TLR7-dependent immune dysfunction plays a vital role in the uncoordinated adaptive immune response in COVID-19 by regulating T-cell and B-cell immunity. Nonetheless, the molecular mechanism of TLR7 signaling in regulating SARS-CoV-2-specific cellular and humoral immunity remains elusive. Understanding the role of TLR7-medited immunity is essential for the elucidation of immunopathological mechanism of SARS-CoV-2 infection and the exploration of therapeutic interventions. Clinical trials or mechanically studies based on primates are warranted to investigate the underlying mechanism of endosomal TLR7 in mediating the adaptive immune response to SARS-CoV-2 infection in the future.

## 4 Mechanisms of TLR7 and the downstream IRF/IFN signaling pathways in regulating SARS-CoV-2 infection

### 4.1 TLRs-mediated IFN signaling activation in regulating autoimmunity

**Figure 1:**
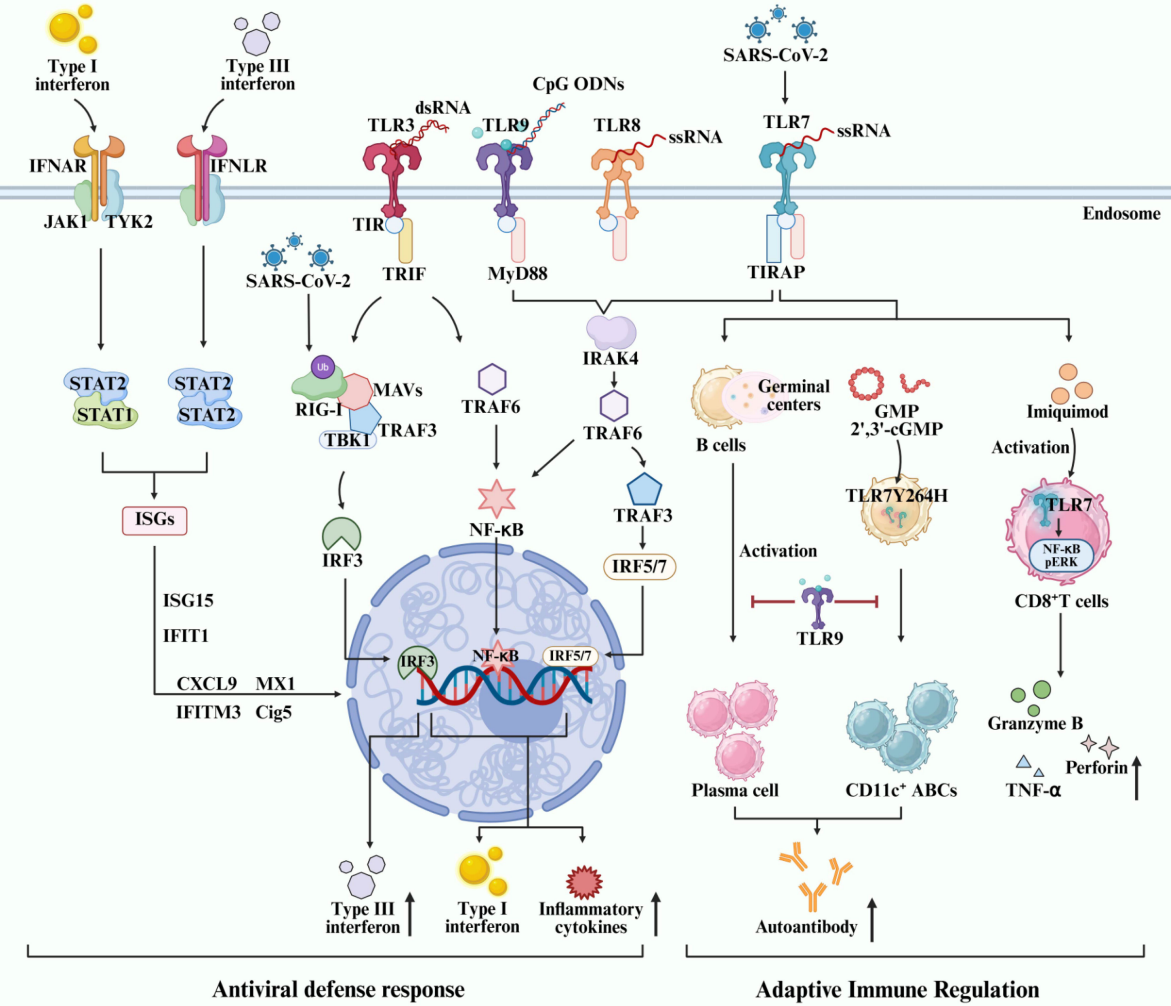
Role and molecular mechanism of endosomal TLRs-mediated IRFs/IFNs signaling activation in regulating autoimmune disorders and antiviral immunity.

TLRs are vital for the initiation and activation of innate immunity, especially for anti-viral immunity. The cell-surface TLRs, such as TLR2 and TLR4, recognize viral protein particles of the virus. The endosomal TLRs of TLR3, TLR7, TLR8 and TLR9 are responsible for the sense the nucleic acid strains or intermediate nucleic acids inside the infected cells by viruses (**Figure 1**). Upon recognition by PAMPs, TLRs undergo a complex activation process characterized by homodimerization or heterodimerization of their ectodomains. This initial step is followed by the dimerization of their intracellular domains, leading to the recruitment of signaling adapters and subsequent activation (Sameer and Nissar, 2021). Distinct TLRs recruit different adaptors but share some downstream regulators in common, such as IRFs and NF-κB, which elicits IFNs and pro-inflammatory cytokine release (Lester and Li, 2014).

#### 4.1.1 TLRs-mediated IFN signaling activation in regulating antiviral immunity

During the last decade, the crosstalks between IRFs/IFN signaling and TLRs signaling have been implicated in various viral infections, such as Influenza A, SARS-CoV-2, and RSV. They induce or antagonize IFNs signaling depending on various factors. The membrane receptor for type I, II and III IFNs signal is IFN alpha receptor (IFNAR), IFN gamma receptor (IFNGR), and IFN lambda receptor (IFNLR), respectively. Activation of TLRs and the IFN signaling cascade leads to enhanced expression of antiviral interferon-stimulated genes (ISGs), ultimately contributing to the establishment of an antiviral state within the host cell. Multiple signaling pathways are engaged in antiviral defense, primarily mediated through two key adapter proteins: MyD88 and IL-1R resistance (TIR) domain-containing adapter-inducing IFN-β (TRIF) (Kosciuczuk et al., 2019; Gallucci et al., 2021; Ji et al., 2023). TLR activation recruits either the MyD88- or TRIF-dependent pathway. MyD88 initiates downstream NF-κB and mitogen-activated protein kinase (MAPK) signaling, leading to pro-inflammatory cytokine production. TRIF is selectively recruited to TLR3 and TLR4. And, TRIF activates TBK1, which phosphorylates IRF3/IRF7, inducing type I IFN production. Subsequent Janus kinase (JAK)-signal transducer and activator of transcription (STAT) signaling is stimulated by IFNs to amplify antiviral gene expression. Additional pathways such as PI3K-Akt-mTOR further modulate immune and metabolic responses during infection. Two TNF receptor-associated factors (TRAFs), namely TRAF3 and TRAF6, can be activated by members of the IRAK family, leading to the production of type I IFN or pro-inflammatory cytokines, respectively (Häcker et al., 2006, 2011). Recent studies have suggested that type III IFN signaling play an important role in influenza virus infection by inducing a more sustained ISGs expression and a non-redundant front-line antiviral response to control viral replication and abundant inflammation through the IFNLR-related signaling in neutrophils and epithelial cells (Galani et al., 2017; Vlachiotis and Andreakos, 2019; Yadav et al., 2023). The heterodimerization of type III IFNLR is insufficient for JAK-STAT activation, while IFN-λ induces a JAK-STAT cascade and the ultimate activation of endogenous type III IFN pathway via JAK1 and TYK2 kinases (**Figure 1**) (Mesev et al., 2023). Type-I and -III IFNs are well documented to defend against SARS-CoV-2 infection, and type-II IFN has also been demonstrated to inhibit SARS-CoV-2 replication in human lung epithelial cells as well as ex vivo human lung tissues through IFNγ-induced IDO1-mediated antiviral response (Yang et al., 2024a). The interference with IFNs and IFN signaling activation is a primary strategy of SARS-CoV-2 to evade the immune recognition, surveillance and clearance of the immune system. The production of autoantibodies against IFNs and loss-of-function variants of type I IFN immunity genes can lead to impaired IFNs antiviral response and ultimately immune evasion of SARS-CoV-2, thereby aggravating COVID-19 or causing PASC (Znaidia et al., 2022; Yao et al., 2023). As a pattern recognition receptor, MDA5 can recognize the RNA of SARS-CoV-2 virus, particularly the dsRNA intermediates generated during viral replication. This recognition triggers downstream signaling via MAVS, leading to the activation of transcription factors IRF3, IRF5, and NF-κB, which subsequently induce the production of IFN-I and IFN-III and mediate antiviral immunity (Yin et al., 2021; Maiti, 2024). RIG-I specifically binds to the 3’ untranslated region (3’ UTR) of SARS-CoV-2 RNA through its helicase domain independently of its C-terminal domain. This mechanism is unique, as it does not stimulate RIG-I’s ATPase activity, thus avoiding excessive immune activation (Yamada et al., 2021). Transfection of RNA from SARS-CoV-2-infected cells into epithelial cells showed that a specific ∼200-nucleotide viral RNA fragment preferentially induces a RIG-I-dependent IFN-I response. This response was significantly reduced in RIG-I-knockout cells, confirming RIG-I as the key sensor for such small viral RNAs and its essential role in initiating innate immunity (Kouwaki et al., 2021). Mechanistically, SARS-CoV-2 antigens suppress IFN response by inhibiting JAK-STAT1 signaling activation (Minkoff and tenOever, 2023), disrupting the RIG-I-MAVS-TRAF3-TBK1 complex (Zheng et al., 2020), impeding RIG-I ubiquitination via tripartite motif 25 (TRIM25) (Wu et al., 2020; Khatun et al., 2023), and reducing IRF3 phosphorylation via Karyopherin alpha 2 (KPNA2) (Xia et al., 2020) (**Figure 1**). These actions collectively suppress the expression of antiviral IFNs and ISGs. Therefore, IFN-based therapy not only establishes a robust antiviral immune state but serves as a promising strategy to counteract SARS-CoV-2 immune evasion. Improving the therapeutic efficacy of IFN-based regimens by increasing the sensitivity of IFNs to SARS-CoV-2 and preventing IFN autoantibodies production as well as IFN gene modifications or editing, remains a key scientific issue to be addressed in the future. Viruses can thwart the endosomal TLRs sensing systems, thereby evading immune surveillance and immune clearance-mediated by TLRs sensing and the antiviral IFN signaling activation. Loss-of-function variants of TLR7 cause immune evasion of SARS-CoV-2 and life-threatening pneumonia among COVID-19 patients, characterized by reduced TLR7-related gene expression, downregulated cytokine-mediated signaling transduction, and subsequent impairment of type I and type II IFN responses (Solanich et al., 2021; Mantovani et al., 2022). Accordingly, TLRs-mediated IFN signaling activation is essential for the antiviral immunity in COVID-19.

#### 4.1.2 TLRs-mediated IFN signaling activation in regulating autoimmune disorders

Currently published studies have extensively investigated the role of TLR7 signaling in humoral and cellular immune responses in autoimmune disorders, particularly regarding B cell-intrinsic TLR7 (**Figure 1**). B cell-intrinsic TLR7 but not TLR3 is required for the formation of germinal center and the production of autoantibodies (Browne, 2011; Soni et al., 2014). A TLR7 gain-of-function variant (TLR7^Y264H^) has been demonstrated to drive B cells activation and CD11c^+^ age-associated B cells accumulation in a B cell-intrinsic manner by selectively sensing guanosine and 2’,3’-cGMP in systemic lupus erythematosus (SLE) (Jackson et al., 2014). Accumulated studies have suggested B cell-intrinsic TLR9 attenuates systemic inflammation and autoimmunity by inhibiting antigen uptake and the processing of activated B cells, affinity maturation, generation of antigen-specific plasma cells, and serum IgG avidity (Jackson et al., 2014; Akkaya et al., 2018). Furthermore, the role of B cell-intrinsic TLR7 and TLR7 signaling in promoting severe SLE has also been well observed in TLR9-deficient mice, highlighting a cis regulatory interplay between the protective B cell-intrinsic TLR9 and the pathogenic B cell-intrinsic TLR7 in B cell immunity (Satterthwaite, 2021; Cosgrove et al., 2023). All these findings have suggested the opposed impact of B-cell intrinsic TLR7 and TLR9 in regulating adaptive immunity. Accordingly, B cell-directed TLR7 antagonism/TLR9 agonism may be a promising strategy to attenuate systemic inflammation and autoimmune disorders by controlling autoantibody production and aberrant adaptive immune response. However, the precise molecular mechanism remains unclear. Additionally, TLR7 is also essential for T cell response and autoimmune activation. The activation of TLR7 in the endosomes of CD8^+^ T cells by Imiquimod drives a remarkable cytokine transcript signature through the NF-κB and pERK signaling pathway in rheumatoid arthritis (RA) (Swain et al., 2022). Accordingly, TLR7 drives autoimmunity by activating both humoral and cellular immunity, making it a potential therapeutic target for autoimmune diseases. However, more future studies are warranted to elucidate the downstream signaling mechanism of TLR7-mediated IFN signaling activation in autoimmune diseases.

### 4.2 TLR7-mediated IFN signaling activation in SARS-CoV-2 infection

Activation of TLRs triggers the expression of IFNs and ISGs, forming a positive feedback loop in antiviral immunity. Currently available data has implicated cell-type specific distribution and activation of TLRs and IFN-related signaling molecules in defending against SARS-CoV-2 infection (Park and Iwasaki, 2020; Manik and Singh, 2022). However, the precise mechanism underlying TLR7 and the downstream IRF/IFN signaling pathways in regulating innate and adaptive immunity in COVID-19 has not been fully understood due to the complex and sometimes contradictory roles of IFN signaling, which can exhibit both protective antiviral and harmful pro-inflammatory effects in different stages of infection. Additionally, the extensive crosstalk between TLR7, other innate immune pathways, and adaptive immune cells creates a highly interconnected network that is difficult to dissect experimentally. TLR7 is a MyD88- and IRAK-4-dependent endosomal ssRNA sensor (**Figure 1**). Patients with inherited MyD88 or IRAK-4 deficiency are predisposed to COVID-19 pneumonia, suggesting the pivotal role of TLR7 signaling-related genes in the onset of severe COVID-19 (Garcia-Garcia et al., 2023). TLR7-MyD88-TIRAP signaling is crucial for the antiviral IFNs response to SARS-CoV-2 infection, whereas disrupting this signaling complex using various TLR7 antagonists and N-acetylcysteine leads to impaired viral RNA sensing and MyD88 signaling (van der Sluis et al., 2022). Most importantly, TLR7-mediated innate immunity plays a central role in the first-line antiviral defense against SARS-CoV-2 infection by activating IFN signaling pathway and enhancing the expression of IFNs and ISGs in immune cells (Jackson et al., 2023). IFN signaling pathway and ISGs play pivotal roles in the antiviral response in COVID-19 (Eskandarian Boroujeni et al., 2022; Song et al., 2023). Transcriptional activation of IFNs and ISGs is extensively observed in SARS-CoV-2-infected cells, such as ISG15, IFIT1, IFITM3, CXCL9, myxovirus resistance 1 (MX1), and Cig5 (**Figure 1**) (Skaug and Chen, 2010; Solimani et al., 2021; Unali et al., 2023). IRF3 and IRF7 are the main IRFs involved in the downstream of TLR signaling events, inducing the production of type I IFNs (Jefferies, 2019). Enhanced production of type I IFNs in DCs can be induced through the activation of TLR7-IRF5 signaling during SARS-CoV-2 infection (**Figure 1**) (Cords et al., 2023). A number of studies have implicated impaired type-I IFNs response due to autoantibodies against IFN-α and IFN-β, agonists of TLR7 or rare variants of key genes in TLR7-IRFs signaling is closely related to severe COVID-19 (Hadjadj et al., 2020; Zhou et al., 2020; Matuozzo et al., 2023). Higher risk of life-threatening COVID-19 is highly associated with anti-IFN-α autoantibody and loss-of-function variants of type I IFN immunity genes depending on TLR7. Therefore, the activation of IFN-related cascade due to dysregulated TLR7 ultimately leads to immune hyperactivation, particularly in severe COVID-19, but lacking well-known signaling mechanism. There might be intricate interplay between TLRs signaling and IRFs/IFNs signaling in regulating inflammation and antiviral immunity due to the obviously dysregulated TLRs and abnormal IFN signaling activation in COVID-19. Nonetheless, the precise signaling mechanism underlying diverse types of TLRs and IFNs underlying different viral infections is different, particularly the endosomal TLR7 and TLR7-mediated IRF/IFN signaling activation. Besides, IFN responses differ between diverse mice strains and between mice and humans. The role of TLR7-mediated IFN signaling activation in COVID-19 warrants to be investigated in more human clinical studies or studies using the SRAS-CoV-2-infected primates animal model. Furthermore, the association between innate immunity and B-intrinsic TLR7-mediated adaptive immune response and the production of antibody in SRAS-CoV-2 infection remains largely unknown. Elucidating the promising therapeutic target(s) in the TLR7-mediated IRF/IFN signaling pathway is critical for the development of therapeutic strategies or vaccines against SARS-CoV-2 infection that aim to modulate the host immune response for more effective viral clearance and prevention of severe COVID-19 or long-COVID.

## 5 SARS-CoV-2-specific adaptive immune dysregulation and the potential association with TLR7 in long-COVID

Currently, a great amount of people is suffering from long-COVID or PASC, whereas the potential biological factors driving post-COVID-19 pathology remain unclear. Some factors been well documented to contributing to PASC pathology, such as microbiome, coagulation, and abnormal neuroimmunity. Little is known of the precise molecular mechanisms underlying long COVID. The crosstalk between adaptive cellular immunity and humoral immunity contributes to long-COVID symptoms, such as sleep disturbance, fatigue, “brain fog”, and osteoarthralgia, by inducing long-sustained inflammation and uncoordinated adaptive immunity (Yin et al., 2024). The study by Yin K and the colleagues has suggested systemic inflammation and persistent T and B cell responses in long-COVID individuals (Yin et al., 2024). They reported elevated ratios of exhausted CD8^+^ T cells and inflamed tissues-infiltrated CD4^+^ T cells alongside with increased SARS-CoV-2 antibodies, suggesting SARS-CoV-2-specific immunity in 8 months post-infected long-COVID individuals. Furthermore, the attenuation of functional polarization of CD8^+^ T cells by blocking granzyme B and IFN-γ is demonstrated to be an effective approach to prevent from PASC and further complications (Wiech et al., 2022). Therefore, long-COVID manifests with sustained inflammation, impaired IFN response, and SARS-CoV-2-specific B and T cell responses. Maintaining the balance of adaptive immunity mediated by B and T cells is essential for controlling IFNs production and long-COVID progression. However, the underlying mechanism of IFN signaling activation in modulating adaptive immunity and its contribution to the establishment of long-term immunity against SARS-CoV-2 infection remain not fully understood. Identifying checkpoints in the upstream or downstream of IFN signaling involved in antiviral immunity is of great importance to explore novel therapeutic strategies or more effective vaccines to prevent PSAC.

Emerging studies has implicated the critical role of intrinsic TLR7 in adaptive immune response to SARS-CoV-2 and pathogen-like COVID-19 vaccines by regulating the activation of IFN signaling as discussed previously (Rossmann et al., 2021; Miquel et al., 2023; Yan et al., 2023; Yin et al., 2023). Little is known about the modifying effect of TLR7 in long-COVID. A previous study has implicated that a loss-of-function hemizygous variant (c.1386_1389dup) of TLR7 is positively associated with the long-term neurological complication of post-COVID-19 individuals, suggesting the potential role of TLR7 in long-COVID (Noor Eddin et al., 2023). Although the significant role of TLR7 and IRF-IFN signaling has also been implicated in antiviral response, the precise mechanism of TLR7-mediated IFN signaling activation in regulating the adaptive immunity against SARS-CoV-2 infection and long-COVID remains largely unknown. Whether and how TLR7-mediated IFN signaling activation contributes to the long-sustained inflammation and uncoordinated adaptive immunity in long-COVID is poorly defined. Long-COVID deeply affects the quality of life for affected individuals. Understanding the nuanced mechanisms underlying TLR7 and IRFs/IFNs signaling can offer valuable insights into the development of new targeted therapies for COVID-19 and highly effective vaccines to prevent from long-COVID.

## 6 Beneficial effects of vaccines and therapeutic strategies targeting TLR7 and IRFs/IFNs signaling in COVID-19

The beneficial effects of vaccines and therapeutic strategies targeting TLR7 and IRFs/IFNs signaling are paramount in combating COVID-19 pandemic and long-COVID. TLR7 activation triggers the innate immune response, leading to the production of IFNs and pro-inflammatory cytokines that help in combating with viral infections. Reversely, by enhancing IRF-mediated induction of IFNs, therapeutic strategies can bolster the antiviral defense mechanisms, dampen excessive inflammation, and promote viral clearance. Therefore, targeting TLR7 and IRFs-IFNs signaling pathways holds potentials in COVID-19 treatment, the establishment of long-term immunity to SARS-CoV-2 infection, and the exploration of highly effective antiviral vaccines.

Some currently available data has been gained from preclinical studies and early-phase clinical trials regarding TLR7-targeting drugs, IRF-targeting drugs and IFN-targeting drugs in COVID-19. Nonetheless, the efficacy and safety estimates for these drugs are still evolving. TLR7 agonists are being explored to boost antiviral immunity, while TLR7 antagonists aim to modulate inflammatory responses. Vesatolimod is a TLR7 agonist holding potentials in enhancing antiviral immunity (SenGupta et al., 2021; Usero et al., 2023). Vesatolimod activates TLR7 to induce the production of IFN-α/β and pro-inflammatory cytokines in immune cells, which enhances the body’s early recognition and response capacity against viruses and suppresses viral replication. Increased expression of ISGs and elevated cytokine release in patients following Vesatolimod treatment, suggesting it may enhance the immune response against SARS-CoV-2 through a similar mechanism (Riddler et al., 2021). But little is known about its therapeutic efficacy in COVID-19. Most importantly, the risk of exacerbating inflammation with TLR7 agonists should be treated seriously. TLR antagonist targeting TLR7/9 or agonist targeting TLR7/8 has also been focused on the regulation of autoimmunity, cancer and viral infections, while their potentials in modulating TLR7 signaling for COVID-19 is unclear yet (Khanmohammadi and Rezaei, 2021; Deshmukh et al., 2024; Wu et al., 2024). However, the primary off-target risk of these targeting agents is the induction of a systemic inflammatory response. The widespread release of IFN-α, TNF-α, and IL-6 can mimic severe viral infections or even cytokine release syndrome (CRS), leading to fever, fatigue, hypotension, and organ toxicity. TLR9 agonist play a crucial role in early antiviral immunity by recognizing viral nucleic acids, activating immune cells, and inducing the production of IFN-I and pro-inflammatory cytokines. However, excessive activation may trigger a cytokine storm, leading to immunopathological damage (Dyavar et al., 2021; Khanmohammadi and Rezaei, 2021). Although TLR9 is not a direct recognition receptor for SARS-CoV-2, its agonists can still indirectly participate in regulating the immune response in COVID-19 by mimicking viral nucleic acids or recognizing damage-associated molecular patterns (DAMPs) released during tissue injury (Khanmohammadi and Rezaei, 2021; Naqvi et al., 2022). TLR9 is widely expressed in various immune cells, such as B cells and plasmacytoid dendritic cells. Its agonists may non-specifically activate multiple cell types, potentially leading to systemic inflammation. For instance, TLR9 activation in B cells has been associated with autoimmune diseases including type 1 diabetes, suggesting a risk of exacerbating autoimmune pathology in conditions such as COVID-19 (Sha et al., 2021; Yang et al., 2024b). Furthermore, studies in obesity models have shown that B cell-specific deletion of TLR9 reduces adipose tissue inflammation, which conversely implies that TLR9 agonists might promote metabolic-related inflammatory pathways (Yang et al., 2024b). In severe COVID-19 patients, monocytes exhibit a state of TLR tolerance (Naqvi et al., 2022), wherein TLR9 agonists may fail to reverse this hypo-responsive condition and could potentially exacerbate immune exhaustion. Furthermore, the synergistic effects between TLR9 and TLR3 in conventional type 1 DCs (cDC1) are differentially regulated by the nuclear receptor co-repressor 1 (NCoR1) (Mishra et al., 2022). Notably, loss of NCoR1 enhances TLR9 responses while attenuating TLR3 signaling, indicating that cell type-specific epigenetic mechanisms may interfere with the targeting efficiency of TLR agonists. Clinical data regarding the efficacy and safety of IMO-9200 in COVID-19 are encouraged in the future. Nonetheless, the significant challenges, such as the potential for disrupting humoral immunity and the complex interplay between TLR7 and TLR9 in autoimmune contexts, should not be ignored. IRFs exert vital effects on the expression of IFNs, ISGs and other immune-related genes. Drugs targeting IRFs, such as inhibitors or modulators, could potentially influence the immune response to SARS-CoV-2 and modulate the host immune response to achieve a balance between viral clearance and immune-mediated tissue damage. Iberdomide (CC-220) is a cereblon-targeting drug that modulates the transcription factor IRF4, thereby playing a role in the context of viral infection (Ne et al., 2022). In clinical trials for SLE, iberdomide significantly reduced the type I IFN gene signature in patients with high baseline expression (Lipsky et al., 2022). Given that severe COVID-19 is also associated with aberrant type I IFN activation (Lipsky et al., 2022), it is hypothesized that iberdomide may mitigate COVID-19-related hyperinflammation through a similar mechanism. Furthermore, iberdomide modulates both innate and adaptive immune responses and remodels the tumor microenvironment, as evidenced in malignancies such as multiple myeloma (Van Oekelen et al., 2024). These immunomodulatory properties align with the need to counteract immune dysregulation observed in severe COVID-19, suggesting its potential applicability in managing COVID-19 immunopathology. GSK2831781, an inhibitor of IRF4, has been investigated due to its ability to modulate IRF-mediated immune response in T-cell-mediated inflammatory and autoimmune diseases, such as ulcerative colitis and psoriasis (Ellis et al., 2021; D’Haens et al., 2023). In the future, early-phase clinical trials may shed light on the efficacy and safety of these drugs in COVID-19 patients. IFNs are signaling proteins involved in antiviral defenses against SARS-CoV-2 by boosting the innate immunity and inhibiting viral replications. Clinical trials have evaluated the efficacy and safety profile of IFN-targeting drugs, whereas the results may vary depending on the specific formulation and patient population. Additionally, a number of factors, such as dosing, patient characteristics, and disease severity, can influence outcomes. Continued research and larger-scale clinical trials are warranted to further assess the potentials of these drugs in treating COVID-19. Overall, the combined approach of vaccination alongside therapeutic interventions targeting TLR7 and IRFs/IFNs signaling presents a multifaceted strategy to mitigate the impact of COVID-19. By harnessing the power of both preventive and treatment modalities, it would be more effective to control the spread of the virus, reduce disease burden, and safeguard public health.

## 7 Conclusions and future directions

Innate immunity is the first line to defend against SARS-CoV-2. The critical role of TLR7-mediated IFN signaling activation in innate immunity has been well documented in defending against various viral infections, including SARS-CoV-2 but lacking sufficient evidence for the signaling mechanism. The intrinsic TLR7 is not only essential for the initiation of innate immune response to SARS-CoV-2 infection but also the regulation of adaptive B cell- and T cell-mediated humoral and cellular immunity. The dual roles of TLR7 in antiviral defense and inflammatory pathology are shaped by the spatiotemporal context and magnitude of its activation. Under controlled conditions, transient TLR7 signaling in pDCs promotes type I IFN production, supporting effective viral control. However, sustained or excessive activation driven by high viral loads or self-RNA exposure triggers hyperinflammatory responses in macrophages and other myeloid cells, leading to pathological cytokine release. This shift is further amplified through synergistic crosstalk with other innate immune pathways (e.g., inflammasome activation, RIG-I/MDA5 sensing) and the failure of endogenous regulatory mechanisms, such as feedback inhibitors or tolerance induction. it exhibits extensive cross-talk with other nucleic acid-sensing pathways. For example, the co-activation of RIG-I enhances antiviral IFN responses but may also exacerbate inflammatory tissue damage if not properly regulated. In contrast, engagement of TLR3, which induces IRF3-driven IFN responses with less inflammatory output, may partially compensate for or modulate TLR7 hyperactivation. Feedback inhibitors also play a central role in shaping TLR7 signaling dynamics, such as IRAK-M, SOCS1, and A20, which attenuate TLR7-mediated NF-κB and IRF7 activation. Consequently, the functional outcome of TLR7 activation is determined by integrated signals from ligand availability, temporal dynamics, cellular microenvironment, concurrent PRR engagement, and the capacity of negative regulatory networks.

While the role of TLR7-mediated IRF/IFN signaling in the adaptive immune response to SARS-CoV-2, particularly in long-COVID, remains mechanistically unclear, its therapeutic potential warrants systematic investigation. To bridge key knowledge gaps, future studies should prioritize innovative approaches such as single-cell transcriptomics and spatial multi-omics within primate models to delineate cell-specific TLR7 responses and immune crosstalk. Clinical trials evaluating TLR7 agonists, IRF modulators, or IFN-based therapies should incorporate precise endpoints including viral persistence, T-cell memory quality, and autoantibody profiles, alongside integrative biomarkers such as TRS-based gene signatures or multiplex cytokine mapping. Furthermore, combining vaccination with targeted immunomodulation of the TLR7-IRF-IFN axis may offer a dual strategy to enhance viral clearance, regulate adaptive immunity, and mitigate long-COVID sequelae. Such focused efforts are essential to advance this pathway from mechanistic insight to therapeutic application.
